# Nitrogen fertilizer affects the cooking quality and starch properties of proso millet (*Panicum miliaceum* L.)

**DOI:** 10.1002/fsn3.3789

**Published:** 2023-11-03

**Authors:** Hongyu Liu, Beibei Liu, Haolu Zhou, Yinghui Huang, Xiaoli Gao

**Affiliations:** ^1^ State Key Laboratory of Crop Stress Biology in Arid Areas, College of Agronomy Northwest A&F University Yangling China; ^2^ Ankang Vocational Technical College Ankang China

**Keywords:** cooking quality, nitrogen fertilizer, proso millet, starch properties

## Abstract

Nitrogen has a critical influence on the yield and quality of proso millet. However, the exact impact of nitrogen on the cooking quality of proso millet is not clear. In this study, the cooking quality and starch properties of two proso millet varieties (waxy‐Shaanxi millet [wSM] variety and non‐waxy‐Shaanxi millet [nSM] variety) were compared and analyzed under nitrogen fertilizer treatment (N150, 150 kg/hm^2^) and a control group without nitrogen application (N0, 0 kg/hm^2^). Compared with the N0 group, the N150 treatment significantly increased protein content, amylose levels, and total yield. Employing rapid visco analyser and differential scanning calorimetry analyses, we observed that under the N150 treatment, the peak viscosity and breakdown viscosity of proso millet powder were diminished, while the setback viscosity and enthalpy values (ΔH) increased. In addition, nitrogen treatment increased the solids content in the obtained rice soup and significantly hardened the texture of the rice. At the same time, we noticed that the absorption capacity of starch in water and oil was enhanced. These results showed that nitrogen fertilizer had significant effects on the cooking quality and starch properties of proso millet.

## Graphical abstract

Nitrogen application significantly affected the cooking quality and starch properties of proso millet. Compared with the control group (N0), the protein, amylose content, and yield of the proso millet were significantly increased, and the soup solids were increased, which made the proso millet easier to boil into porridge. The final viscosity, setback value, initial temperature (To), peak temperature (Tp), and enthalpy value (∆H) of proso millet powder increased significantly, but the PV, BV, and BD decreased. The adhesiveness of proso millet rice decreased, the texture was harder, the water absorption and oil absorption of proso millet starch increased, and the solubility and swelling power decreased.

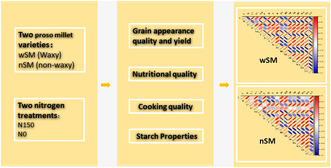

## 1 INTRODUCTION

Proso millet (*Panicum miliaceum* L.) is an ancient crop that originated in China. It belongs to the genus Gramineae and has a cultivation history of more than 10,000 years (Li et al., 2020). This adaptable crop has been widely distributed on various continents, including the Americas, Europe, and Asia. Due to its remarkable endurance against adverse soil conditions, salinity, and drought, coupled with its abbreviated reproductive cycle, proso millet remains a major food in arid and semiarid Chinese regions (Habiyaremye et al., 2017). Recent research has highlighted the potential of proso millet as an environmentally friendly cultivation candidate for improving salinized soils, making it an appropriate synonym for “halophyte” (Yuan et al., 2022). Proso millet is called yellow rice after peeling, and it is rich in protein, starch, amino acids, and mineral elements. It also contains natural active substances with hypoglycemic, hypolipidemic, and antioxidant functions, such as phenolics and phytic acid (Hegde & Chandra, 2005). Notably, its gluten‐free nature positions yellow rice as an optimal resource for producing items catering to individuals with celiac disease as well as infants. In addition, by cleverly mixing with wheat flour, this versatile grain can be shaped into a staple food or a casual dietary choice while improving processing characteristics (Zarnkow et al., 2007). Beyond its nutritional bounty, yellow rice encapsulates an array of health‐promoting attributes, including its capacity to thwart atherosclerosis (Singh et al., 2011). With the improvement of living standards and the pursuit of dietary balance, the demand for proso millet is gradually pivoting from sheer yield to quality. Therefore, investigations of quality standards, cultivation of palatable cultivars, and breeding of premium‐grade strains have emerged as key pathways for proso millet research (Pilat et al., 2016).

In addition to complex genetic susceptibility, the quality of crops is essentially determined by a confluence of factors such as soil composition, climatic nuances, and cultivation methodologies throughout their growth cycle (Falade et al., 2014). Nitrogen is an important component of crop growth and development trajectory; the judicious application of nitrogen fertilizers is a key agronomic strategy to improve yield and quality within the agricultural domain (Zhu et al., 2016). The quality of proso millet mainly includes appearance, nutrition, and cooking quality. Grain shape and color are the main aspects of appearance quality; protein content is the main indicator of its nutritional value; and cooking quality is intricately linked to viscosity properties (Wang et al., 2023). The main properties of viscosity are peak viscosity, final viscosity, and breakdown viscosity, which collectively reveal the cooking characteristics of the grain (Fan et al., 2017). Distinguished by its amylose content, proso millet can be divided into waxy (low amylose content) and non‐waxy (high amylose content), and there are significant differences in cooking quality (Yang et al., 2018). Recent studies on rice have revealed that nitrogen treatment has an inhibitory effect on the peak viscosity of both rice flour and starch, precipitating an intricate modulation of rice's edibility for the structural transformations within the protein and starch matrices during the cooking process (Shi et al., 2023). Zhou et al. (2020) found that nitrogen application increased the proportion of short amylopectin (DP6‐12), decreased the proportion of long amylopectin (D ≥ 37), and decreased the setback viscosity and pasting temperature (PT) of rice. Furthermore, Xiong, Chen, et al. (2022) and Xiong, Tan, et al. (2022) analyzed the multi‐level structure, physicochemical properties, and texture of Indian rice starch under six nitrogen fertilizer treatments. It was found that the thermal stability and gelatinization properties of rice starch increased under nitrogen fertilizer treatment, and the texture of rice was improved. In general, the effect of nitrogen fertilization on crop cooking quality was mainly focused on bulk crops such as rice in previous studies, but scant attention was paid to how nitrogen application affects the cooking and eating quality of proso millet systematically. Therefore, two proso millet varieties were used in this study to investigate the effect of nitrogen fertilization on the cooking quality and starch properties of proso millet. Our study includes a comprehensive assessment, encompassing the grain appearance, basic components, yield, pasting, and thermal properties of flour, textural properties of rice, water, and oil absorption, freeze–thaw stability, light transmittance, dissolution, and swelling power of starch. Our objective was to reveal the effects of nitrogen fertilizer on proso millet, which was of critical importance not only for the cooking quality and starch properties of proso millet but also for its breeding.

## 2 MATERIALS AND METHODS

### 2.1 Plant materials and experimental design

The experiment was conducted in 2022 at the Yulin Academy of Agricultural Sciences (38°22′ N, 109°44′ E) in Shaanxi Province. Waxy‐Shaanxi millet No. 1 (wSM) variety and non‐waxy‐Shaanxi millet No. 2 (nSM) variety were used as plant materials, and urea was applied as nitrogen fertilizer (total nitrogen (N) ≥ 46%). The soil type was sandy loam, and 0–20 cm of soil was taken to determine soil nutrients according to the method of Zhang et al. (2020). Soil nutrient content was 9.6 g/kg of organic matter, 0.62 g/kg of total nitrogen, 0.61 g/kg of total phosphorus (P), 19.43 g/kg of total potassium (K), and pH 8.27. The experiment was carried out in a randomized group design with three repeats. The plot area was 20 m^2^ (4 m × 5 m) with 40 cm of row spacing. The nitrogen treatment group (N150) was treated with 150 kg/hm^2^ of nitrogen, while the control group (N0) was not treated with nitrogen. Fertilizer for nitrogen treatment was applied uniformly to the soil of each plot before the swing, and field management was carried out by local management practices.

### 2.2 Grain appearance, quality, and yield

The length (L), width (B), and ratio of length to width (L/B) of the hulled grains were measured using an automatic seed‐counting and analyzing instrument (Model SC‐G; Wanshen Ltd., Hangzhou, China) (Li et al., 2015).

The color of the hulled grains was assessed using a chroma meter (Colorimeter Ci7600; Aisaili Color Technology Inc.) (Falade & Christopher, 2015). The color was characterized using variables L*, a*, and b*, where L* represents a range from black (0) to white (100), a* represents a spectrum from red (+) to green (−), and b* represents a range from yellow (+) to blue (−). The total color difference level (∆E*) and color intensity (∆C*) of the hulled grains were calculated using the following equations:

(1) $$∆E*=\sqrt{{\left(∆L*\right)}^{2}+{\left(∆a*\right)}^{2}+{\left(∆b*\right)}^{2}}$$

(2) $$∆C*=\sqrt{{\left(∆a*\right)}^{2}+{\left(∆b*\right)}^{2}}$$

All plants in each plot were harvested and the grains were hulled, the actual yield of the plot was weighed and recorded, and finally converted to hectare yield after the proso millet matured.

### 2.3 Nutritional quality of the flour

Proso millet grains were hulled and ground with universal high‐speed smashing machines (FW100; Taisite Ltd.) and sieved with a 100‐mesh sieve to obtain flour.

The amylose content and total starch content were measured by using an amylose content kit (BC4265; Solarbio Science & Technology Co.) and a starch content kit (BC0700; Solarbio Science & Technology Co., Ltd.) according to the manufacturer's instructions.

The protein content of the flour was determined via the Kjeldhal method, using a protein‐nitrogen coefficient of 6.25. The protein fractions were extracted according to Mao et al. (2014), with slight modifications. The solutions of albumin, globulin, prolamin, and glutelin were extracted using distilled water, 0.5 moL/L NaCl, 75% ethanol, and 0.05 moL/L NaOH in turn. The protein fraction content was measured in the same way as the protein content method mentioned before.

The crude fat content (CFC) of proso millet was determined by the residual method. 2.0 g (M) proso millet powder was wrapped with filter paper and dried in a 105°C oven for 3 h, then weighed as M1 after being cooled in a dryer. The samples were packed into the soxhlet fat extractor, and soaked with anhydrous ether completely for more than 16 h. The soaked sample is put into an extraction bottle, and several zeolites are added. Anhydrous ether was reintroduced into the extractor, all parts of the apparatus were connected, condensed water was connected, and a 60–75°C water bath was heated to make the ether reflow with an 8 times per hour reflow frequency. After 8 h, the sample package was placed in a fume hood to make the ether evaporate, oven‐dried at 105°C for 2 h, and weighed as M2 after cooling. The CFC is calculated as follows:

(3) $$\mathrm{CFC}\;\left(\%\right)=\left(M1–\text{M2}\right)\times 100/M$$

### 2.4 Cooking quality

#### 2.4.1 Cooking properties

After washing, 2 g (m1) of proso millet grains and 30 mL of water were boiled for 30 min and left at room temperature for 30 min until cooled. The pH of the proso millet soup was measured using the TE20 pH meter (Mettler Toledo Instrument Company). After the rice soup was fixed to 45 mL with distilled water, 10 mL of the supernatant was poured into an aluminum box and dried at 105°C to a constant weight to obtain the dry matter weight of the rice soup. The absorbance at 660 nm by spectrophotometer was measured as the iodine blue value, which used the volume of 1 ml supernatant, 1 ml iodine reagent and 5 ml HCl was determined to 100 ml by distilled water. The transmission of light was measured at 660 nm. The weight of the cooked grains (m2) was measured after the lower layer of the grains was transferred to the gauze and left at room temperature for 1 h (Yang et al., 2018). The water absorption rate (WAR) was calculated as follows:

(4) $$\mathrm{WAR}\;\left(\%\right)=\left(m2–m1\right)\times 100/\text{m1}$$

#### 2.4.2 Pasting properties

The pasting properties of proso millet were determined using the RVA 4500 Rapid Visco Analyzer. A 14.0% moisture sample was taken at 3 g with 25.0 mL of distilled water added to a total weight of 28.0 g, and then the sample was placed in a heating table for determination using the following program parameters: the sample was held at 50°C for 1 min, the temperature was increased to 95°C within 3.7 min and held for 2.5 min, and then the temperature was decreased to 50°C in 3.5 min, and finally 50°C was kept for 2 min (Zhu et al., 2010).

#### 2.4.3 Thermal properties

Referring to the method of Wang et al., the thermal properties of starch were studied by differential scanning calorimetry (DSC, Q2000, USA). 3 mg sample and 6 μL of pure water were sealed in the crucible and stored overnight in a 4°C refrigerator. The test temperature is 30–100°C at a rate of 10°C/min (Wang et al., 2020).

#### 2.4.4 Textural properties

Freshly cooked proso millet rice was analyzed by the TA‐XT plus texture analyzer (Stable Micro Systems Ltd), according to Xia et al. (2017). PO 5 mm probe was used to carry out a double‐cycle compression test with a 70% compression ratio. The probe height is set to 30 mm, 10 g triggering force, at a speed of 0.5 mm/s.

### 2.5 Starch properties

#### 2.5.1 Starch extraction

200 g proso millet grains were hulled using a high‐speed mill, and the powder was sifted by a 80‐mesh sieve. Then a 0.2% NaOH solution was added to 100 g of proso millet powder at a rate of 1:10 (g/mL). Starch suspension was obtained after being bathed in water in a 30°C pot overnight, then the mixture was centrifuged at 4000 r/min for 10 min after sifting using a 200‐mesh sieve. The supernatant was discarded, the gray‐green impurities were scraped off with a small key, and then washed with distilled water repeatedly three times until the removal of impurities left only white precipitation. The precipitate was mixed with distilled water and pH adjusted to 7 with 0.2 moL/L HCl. After centrifugation, the precipitate was dried in an oven at 40°C and sifted with a 100‐mesh sieve before being stored in a 4°C refrigerator for further use (Gao et al., 2016).

#### 2.5.2 Light transmittance

Referring to the method of Gao et al. (2021), 1 g/mL of starch emulsion was put into boiling water for 30 min, and the starch was completely gelatinized during heating with shaking once every 5 min. After gelatinization, it was left to cool naturally to room temperature. The light transmittance was measured by using a visible‐light spectrophotometer at 620 nm.

#### 2.5.3 Absorption capacities of water and oil

Referring to the method of Singh et al. (2022), the water absorption capacity (WAC) and oil absorption capacity (OAC) of proso millet starches were measured following the method below. 100 mg of starch sample and 1 mL of distilled water/edible oil were mixed fully, centrifuged at 4000 r/min for 15 min, then precipitated and weighed.

#### 2.5.4 Freeze–thaw stability

According to the Zhu et al. (2008) method, 25 mL of 6 g/100 mL of starch milk was boiled in boiling water for 20 min, cooled to room temperature and weighed, then frozen for 24 h, and thawed naturally. Then the precipitate was centrifuged at 3000 r/min for 20 min 0.0.9 Water solubility and swelling power.

Solubility and swelling were measured according to the method of Yu et al. (2020); 0.3 g of starch sample (W) was dissolved into a 3% solution by distilled water in a centrifuge tube, then bathed in water at 50°C, 60°C, 70°C, 80°C, and 90°C for 30 min with constant shaking. Next, the tubes were cooled to room temperature and centrifuged at 3000 r/min for 20 min. The supernatant was put into an aluminum box and dried to a consistent weight (W1). The precipitate in the tube was also dried to a constant weight (W2). The solubility of the proso millet starch was calculated as W/W1 × 100%; the swelling degree of the proso millet starch was calculated using the formula W2/(W − W1) × 100%.

### 2.6 Statistical analysis

One‐way ANOVA and Duncan multiple comparisons were performed using IBM SPSS Statistics 25, and Origin 2021 was used for mapping.

## 3 RESULTS AND DISCUSSION

### 3.1 Appearance quality, nutritional quality, and yield

**FIGURE 1:**
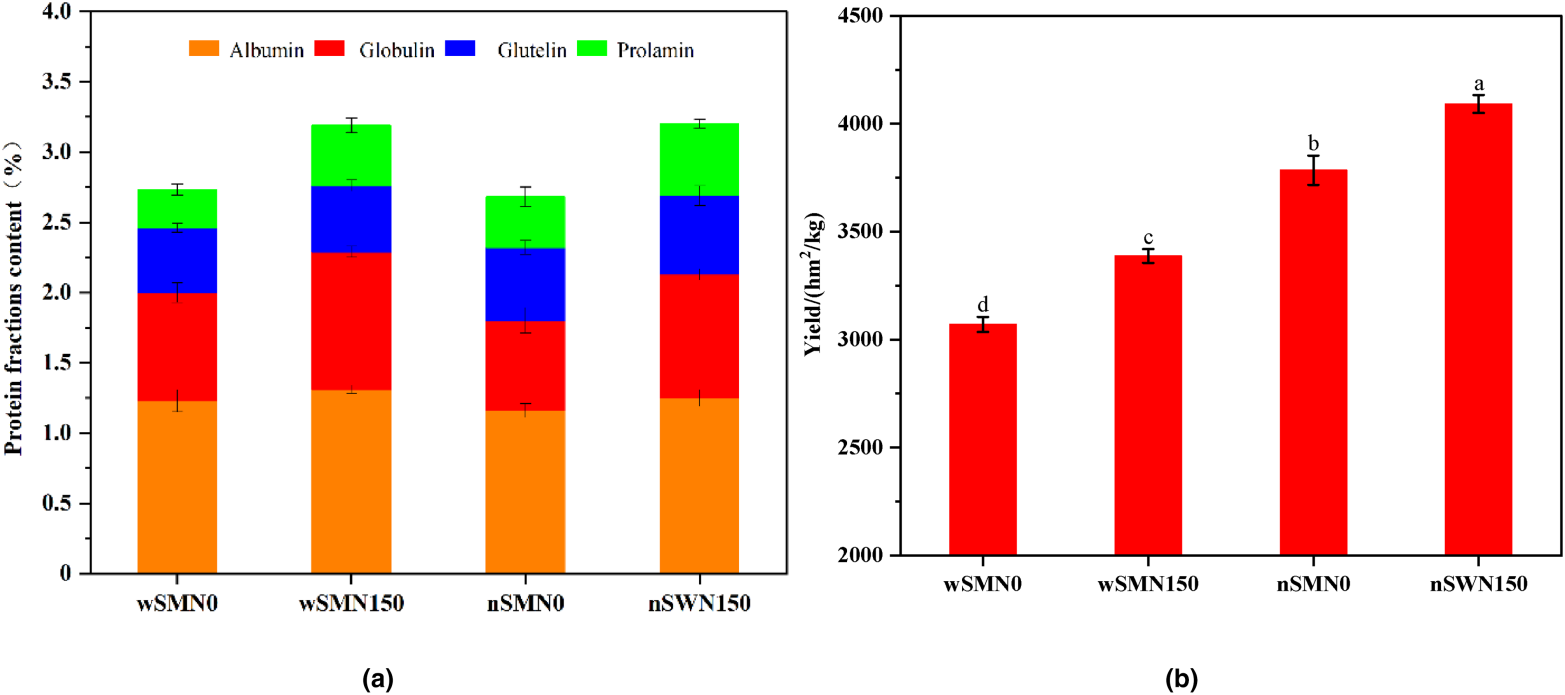
Protein functions content (a) and yield (b) of wSM and nSM under nitrogen treatment. Different letters (a, b, c, and d) represented significant differences (*p* < 0.05).

The ramification effect of nitrogen fertilizer treatment on the appearance quality, nutritional value, and yield of proso millet is detailed in Table 1 and represented in Figure 1. Appearance quality, including shape, size, and color, is an important aspect of grain quality (Zhang et al., 2017), which has many effects on consumers' preferences and the cooking ability of grains (Zhang et al., 2016). As physical properties of grains, length (L), width (B), and aspect ratio (L/B) play a key role in the aesthetic allure and agricultural yield appearance. Overall, it was clear that nitrogen supplementation led to a significant reduction in grain length and width. Notably, there were significant differences between waxy and non‐waxy proso millet, suggesting that amylose content may be correlated with the size of grains. The L*, b, ∆C, and ∆E of the wSM grain were significantly increased under nitrogen treatment; however, the color of the nSM grain did not change obviously. The b* values of nSM were significantly higher than wSM, which is consistent with a study on common buckwheat (Gao et al., 2022). The differences in grain color were mainly between varieties; wSM showed a greater a* value, while nSM showed greater L*, b*, ∆C, and ∆E values. Therefore, there are obvious differences in the color of nSW and wSM grains. The amylose, total starch content, protein content, and yield of proso millet were increased by using nitrogen fertilizer. Remarkably, wSM showed more sensitivity to nitrogen fertilizer and increased its amylose content, protein content, and yield by 120.29%, 24.92%, and 10.33%, respectively. Conversely, nSM showed excellent yield dynamics. Glutamine synthetase (GS), nitrate reductase (NR), glutamic pyruvate transaminase (GPT), and glutamate synthetase (GOGAT) are essential enzymes for protein synthesis (Wang et al., 2016). Nitrogen fertilizer can increase the level of nitrogen metabolism by enhancing the activities of key enzymes in nitrogen metabolism, which culminates in the amplification of grain protein content and resultant yield (Ren et al., 2023).

**TABLE 1:** L value, B value, L/B value, L*, a*, b*, ∆C, ∆E, amylose, crude fat, and protein content of wSM and nSM under nitrogen treatment^a^.

Treatments	L (mm)	B (mm)	L/B	L*	a*	b*	∆C	∆E	Amylose (%)	Total starch (%)	Crude fat (%)	Protein (%)
wSMN0	3.83 ± 0.02b	3.00 ± 0.01a	1.28 ± 0.01c	47 .00 ± 0.78c	14.03 ± 0.54a	15.19 ± 0.19a	20.68 ± 0.49c	51.35 ± 0.59c	3.50 ± 0.16d	62.30 ± 1.27d	3.54 ± 0.04a	9.83 ± 0.16b
wSMN150	3.91 ± 0.04a	2.93 ± 0.01b	1.33 ± 0.03b	48.45 ± 0.68b	14.60 ± 0.71a	15.89 ± 1.06b	21.58 ± 1.25bc	53.04 ± 1.13b	7.71 ± 0.16c	69.45 ± 0.98c	3.23 ± 0.02b	12.28 ± 0.22a
nSMN0	3.79 ± 0.06c	2.75 ± 0.01c	1.38 ± 0.01a	58.68 ± 0.62a	10.21 ± 0.66b	21.62 ± 0.98a	23.91 ± 1.16a	63.37 ± 0.72a	28.47 ± 1.04b	70.15 ± 1.25b	3.17 ± 0.05b	9.91 ± 0.17b
nSMN150	3.76 ± 0.02d	2.73 ± 0.01d	1.37 ± 0.02a	59.26 ± 0.42a	10.11 ± 0.27b	21.02 ± 0.84a	23.33 ± 0.87ab	63.69 ± 0.61a	31.92 ± 0.66a	74.42 ± 0.87a	3.03 ± 0.06c	12.16 ± 0.21a

^a^ Data are means ± SD, *n* = 3. Values in the same column with different letters are significantly different (*p* < 0.05).

The protein component content increased synergistically with the protein content. In our study, albumin content was most abundant and gliadin content was the lowest, which was different from the previously reported results (Wang et al., 2021). This may be due to differences in variety and extraction and purification methods (Akharume et al., 2020). A discernible reduction in the CFC of proso millet after nitrogen application, wSM, and nSM decreased by 8.7% and 4.5%, respectively. The realm of carbon and nitrogen metabolism within crops intertwines via shared requisites such as ATP and carbon frameworks. Competition for ATP and carbon skeletons is generally consistent with the rate of protein synthesis (Zhu et al., 2016). Therefore, the increase in protein content may lead to a simultaneous decrease in CFC. Evidently, the realms of carbon and nitrogen metabolism within crops intertwine via shared requisites such as ATP and carbon frameworks. This metabolic interaction is consistent with the rhythm of protein synthesis (Zhu et al., 2016). Therefore, an increase in protein content may lead to a simultaneous decrease in CFC.

### 3.2 Cooking properties

#### 3.2.1 Cooking characteristics

Cooking characteristics directly affect the popularity of proso millet products on the market. The effects of nitrogen application on the cooking properties of proso millet are shown in Table 2. The water absorption ratio, rice soluble solid, and iodine blue value of proso millet were significantly increased under nitrogen application. However, the light transmittance of rice soup was decreased significantly using nitrogen fertilizer, which is related to the content of soluble solids in the rice soup. As soluble solids accumulate during the cooking progression, the resultant rice soup gains turbidity, resulting in a perceptible diminution in light transmittance, which indicates that nitrogen fertilizer can instigate an augmented dissolution of constituents from proso millet grains into the cooking medium, thus proso millet is easier to boil porridge and convenient for patients with dysphagia to eat (Shim & Lim, 2013). Compared with wSM, nSM showed higher characteristics in water absorption, soluble solids content, and iodine blue value. Rice soup was rich in starch, and nSM had more amylose than wSM, so nSM had higher iodine blue values. Furthermore, nSM displayed a comparatively diminished pH value, a tendency potentially attributed to its elevated concentration of polysaccharides, fatty acids, and protein moieties, which aligns seamlessly with the findings articulated by Yang et al. (2018).

**TABLE 2:** Water absorption ratio, soluble solid, pH, light absorption value, and iodine blue value of wSM and nSM under nitrogen treatment^a^.

Treatment	Water absorption ratio (%)	Soluble solid (mg/g)	PH	Light absorption value (660 nm OD)	Iodine blue value (660 nm OD)
wSMN0	368 ± 47c	62.5 ± 3.8d	6.19 ± 0.06a	67.20 ± 1.08a	0.25 ± 0.01d
wSMN150	443 ± 5b	71.9 ± 3.80c	6.38 ± 0.43a	57.90 ± 1.44b	0.27 ± 0.02c
nSMN0	457 ± 11b	85.3 ± 6b	5.19 ± 0.02b	68.23 ± 3.55a	0.33 ± 0.01b
nSMN150	536 ± 15a	93.5 ± 3a	5.21 ± 0.08b	59.57 ± 2.84b	0.39 ± 0.01a

^a^ Data are means ± SD, *n* = 3. Values in the same column with different letters are significantly different (*p* < 0.05).

#### 3.2.2 Pasting properties

Pasting properties constitute a pivotal theoretical underpinning for grain processing, simultaneously serving as a paramount index for discerning grain quality. Traditionally, the assessment of these attributes has been measured by a rapid viscosity analyzer. Nitrogen application significantly affected the pasting properties of proso millet powder (Table 3). The final viscosity (FV) and setback value (SB) of wSM and nSM were increased, in contrast with peak viscosity (PV), trough viscosity (TV), and breakdown value (BD) being decreased under nitrogen treatment. It is worthy of note that the pasting properties of wSM exhibit a heightened sensitivity to nitrogen supplementation. PV is the maximum viscosity during pasting, which reflects the range of dissolution swelling of starch granules and is related to the water absorption capacity of the starch during heating (Zhu & Cui, 2020). In this study, PV was significantly reduced by using nitrogen fertilizer, indicating that nitrogen application reduces the ability of proso millet powder to bind with water. TV is an important factor in determining the direction of food processing and reflecting the shear resistance of grains at high temperatures (Jiang et al., 2020). In our study, TV exhibited a discernible contraction consequent to nitrogen application, suggesting that nitrogen application reduces the shear resistance of proso millet. BD reflects the resistance to heating; higher BD means lower resistance to heating (Kong et al., 2015). Further insights can be obtained from the SB, which encapsulates the retrogressive tendency within starch pasting dynamics (Gao et al., 2016; Kong et al., 2015). In addition, regeneration affects the quality of starch‐based products derived from yellow rice, which may shorten their shelf life (Li et al., 2017). Within the scope of our investigation, BD was decreased and SB was increased after nitrogen application; therefore, nitrogen application increases the stress tolerance and enhances the retrograde tendency of proso millet powder, mirroring the results of this study in the research of Wang et al. (2023). All indices of pasting properties of nSM were higher than wSM, which is consistent with previous studies on rice (Chung et al., 2011) and maize (Huang et al., 2015), where amylose content was found to exert a positive correlation with TV, FV, SB, PT, and pasting time (PTM).

**TABLE 3:** PV, BV, BD, FV, SB, PT, and PTM of wSM and nSM under nitrogen treatment^a^.

Treatment	PV (cP)	TV (cP)	BD (cP)	FV (cP)	SB (cP)	PT (°C)	PTM (min)
wSMN0	899 ± 43c	420 ± 31b	479 ± 22c	551 ± 28d	131 ± 9d	78.80 ± 0.64b	3.84 ± 0.04b
wSMN150	443 ± 5d	361 ± 10c	349 ± 17d	757 ± 20c	396 ± 28c	78.33 ± 0.03b	3.89 ± 0.04b
nSMN0	1879 ± 18a	679 ± 8a	1200 ± 57a	2799 ± 15b	2150 ± 25b	91.30 ± 0.80a	5.46 ± 0.07a
nSMN150	1563 ± 10b	660 ± 9a	903 ± 7b	2898 ± 19a	2238 ± 29a	91.32 ± 0.75a	5.47 ± 0.07a

^a^ Data are means ± SD, *n* = 3. Values in the same column with different letters are significantly different (*p* < 0.05).

#### 3.2.3 Thermal properties

The effect of nitrogen application on the enthalpy of prosomillet powder is shown in Table 4. Nitrogen application exerts a significant influence on the enthalpy of proso millet. Enthalpy is used to measure the loss of double helix and crystal structure during gelatinization, reflecting the quality and quantity of starch crystallinity (Zhu et al., 2016). The enthalpy values (∆H) of wSM and nSM were increased by 9.8% and 25.5%, respectively, under nitrogen treatment, which means that proso millet powder became insoluble and needed more energy to be dissolved, suggesting that nitrogen application could promote proso millet to form a more stable structure.

**TABLE 4:** Initial temperature (To), peak temperature (Tp), end temperature (Tc), and enthalpy value (∆H) of wSM and nSM under nitrogen treatment^a^.

Treatment	To (°C)	Tp (°C)	Tc (°C)	∆H (J/g)
wSMN0	64.77 ± 1.80bc	77.30 ± 3.20ab	81.53 ± 1.94a	6.10 ± 0.30a
wSMN150	71.03 ± 2.33a	80.50 ± 4.70a	86.10 ± 3.00a	7.03 ± 0.47a
nSMN0	63.93 ± 2.25c	72.30 ± 3.40b	76.30 ± 2.46b	4.50 ± 0.60b
nSMN150	69.70 ± 4.30ab	75.90 ± 1.25ab	82.83 ± 2.25a	6.70 ± 0.90a

^a^ Data are means ± SD, *n* = 3. Values in the same column with different letters are significantly different (*p* < 0.05).

#### 3.2.4 Textural properties

The main principle of the texture analyzer is to simulate the mechanical movements of the mouth during chewing, which stands as a superior alternative to subjective evaluations conducted by human sensory panels, thus elevating objectivity in the assessment of gastronomic attributes (Xiong, Chen, et al., 2022; Xiong, Tan, et al., 2022). The steaming of proso millet is a complex process that includes hydration, crack formation, swelling, leaching, and pasting (Tamura & Ogawa, 2012). Compared with cohesiveness and chewiness, hardness and adhesiveness are relatively consistent indexes of texture properties (Li et al., 2016). The effect of nitrogen application on the textural properties of proso millet is shown in Table 5.

**TABLE 5:** Hardness, adhesiveness, cohesiveness, and chewiness of wSM and nSM under nitrogen treatment^a^.

Treatment	Hardness (g)	Adhesiveness (g/m)	Cohesiveness	Chewiness
wSMN0	483.26 ± 8.76d	−1.39 ± 3.20a	0.28 ± 0.02b	80.74 ± 1.04c
wSMN150	558.79 ± 7.51c	−1.83 ± 0.05b	0.33 ± 0.01a	71.43 ± 0.85d
nSMN0	642.92 ± 3.28b	−3.32 ± 0.04c	0.18 ± 0.01c	123.97 ± 1.51a
nSMN150	672.87 ± 3.54a	−4.12 ± 0.01d	0.17 ± 0.01c	115.62 ± 1.24b

^a^ Data are means ± SD, *n* = 3. Values in the same column with different letters are significantly different (*p* < 0.05).

The hardness of wSM and nSM increased by 15.5% and 6.4%, respectively. In stark contrast, both adhesion and chewiness were significantly reduced compared with the control group (N0). Specifically, adhesion and chewiness attributes of wSM and nSM declined by 31.7% and 11.5%, 24.5% and 6.8%, respectively. Hence, the application of nitrogen engenders an observable transformation in the textural attributes of proso millet rice, accentuating its hardness and concurrently compromising its textural integrity. Some studies have proved that amylose content is an important character that determines the cooking and eating quality of rice and is key to the softness, adhesiveness, and cohesiveness of rice (Li & Gilbert, 2018). Notably, judiciously restrained amylose content within a certain parameter range coalesces to foster the emergence of an elevated taste quality in the rice.

### 3.3 The impact of amylose content and protein content on proso millet cooking quality

As shown in Table 1, the application of nitrogen fertilizer significantly enhanced the amylose and protein content of proso millet. Noteworthy trends emerge in the amylose content hierarchy, observed as nSMN150 > nSMN0 > wSMN150 > wSMN0; parallelly, protein content follows the sequence wSMN150 > nSMN150 > wSMN0 > nSMN0.

According to Table 2, it can be observed that the water absorption ratio, the soluble solid, and the iodine blue value of proso millet rice all increase in correspondence with the elevation of amylose content. Amylose makes a uniform and compact internal grain structure, effectively entrapping large molecular substances and internal moisture (Min et al., 2018), which leads to an augmentation in the water absorption rate of proso millet rice. Meanwhile, the addition of amylose during cooking can lead to an increase in iodine blue value and solubility. Notably, the susceptibility of waxy proso millet grains to rupture becomes accentuated, consequently contributing to the rise in soluble solids in response to elevated amylose content. With the increase in amylose content, the hardness of rice increased, the adhesiveness decreased, and the chewability deteriorated (Table 5), which is consistent with the findings of Wang (2014). During cooking, varieties with higher amylose content liberate more starch, adhering to the rice and forming a thin film that contributes to increased hardness (Li et al., 2017).

The higher the protein content, the more densely it fills the starch. Simultaneously, protein and starch compete for water absorption, which hinders the full expansion of starch and reduces the degree of gelatinization. Fitzgerald et al. (2003) demonstrated that during the pasting, the “denatured protein gel matrix” formed by protein provides mechanical support to starch granules, suppressing their swelling, enhancing their integrity, limiting their rupture, and attenuating starch gelatinization. In Table 5, relative to lower protein content treatments, higher protein content exhibits higher initial temperature (To), peak temperature (Tp), end temperature (Tc), and enthalpy value (∆H), which indicates that a higher protein content makes the flour less prone to gelatinization. This phenomenon might be attributed to the formation of protein‐starch complexes (Wang, 2016), which require more energy to break this association.

Peak viscosity is a sign of starch swelling or bound water, which is related to the characteristics of cooked starch, thus inherently affecting the quality of the final product. The trough viscosity measures the heat resistance and shear strength of starch, reflecting the stability of the starch paste during cooking. Setback viscosity portrays the retrogradation properties of the starch paste (Kong et al., 2015). High BV and low SV indicate better cooking quality, which means post‐cooking retrogradation and firming tendencies are abated (Asante et al., 2013). Therefore, the decreased BV and increased SV of proso millet after nitrogen fertilizer treatment denote a diminished culinary quality. In this study, nitrogen application significantly increased the protein and amylose content of proso millet, which had an impact on cooking quality.

### 3.4 Starch properties

#### 3.4.1 Absorption capacities of water and oil

**FIGURE 2:**
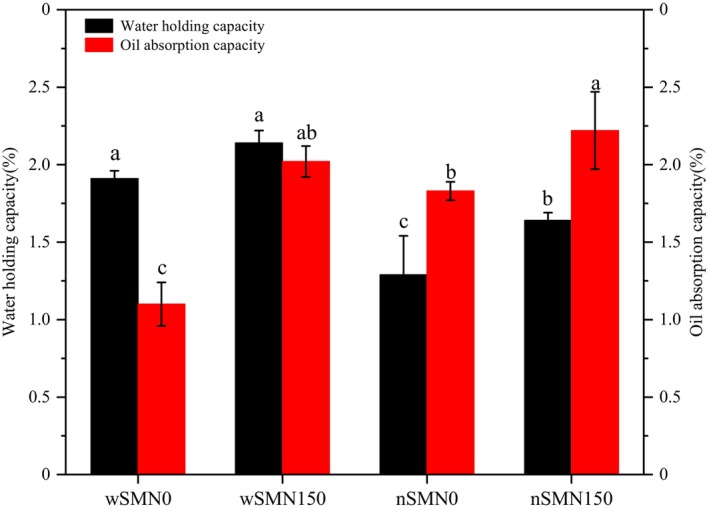
Absorption capacities of water and oil of wSM and nSM under nitrogen treatment. Different letters (a.b.c.d) represented significant differences.

The water and oil absorption of starch reflects the ability of starch to bind with water or oil. As shown in Figure 2, proso millet starch displayed a marked augmentation in both water and oil absorption after nitrogen application, which indicates the substantial impact of nitrogen fertilization on the inherent potential of water or oil molecules to establish hydrogen bonding interactions with the hydroxyl groups that are readily exposed within the crystalline precincts of starch (Gao et al., 2022).

#### 3.4.2 Light transmittance and freeze–thaw stability

**FIGURE 3:**
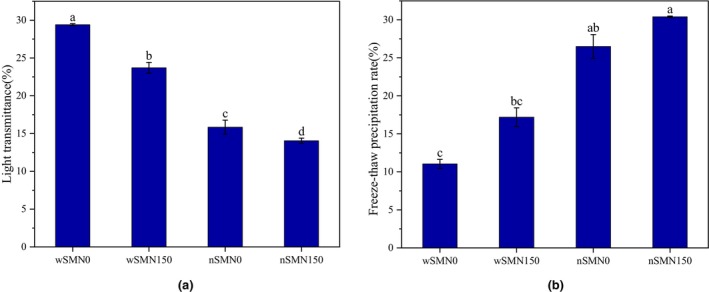
Light transmittance (a) and freeze–thaw precipitation rate (b) of wSM and nSM under nitrogen treatment. Different letters (a, b, c, and d) represented significant differences.

Light transmittance is one of the important external characteristics of starch pastes, which reflects the ability of starch to dissolve and disperse in water (Zhang et al., 2019). Heightened light transmittance conveys augmented dispersion potential, a factor paramount in shaping the characteristics and practical utility of starch‐based commodities, consequently influencing the palatability of food products. As shown in Figure 3, the transparency of proso millet starch paste was significantly reduced following nitrogen application. Furthermore, the transparency of the wSM starch paste is significantly higher than that of the nSM starch paste, which is consistent with previous research results. It has been well established that a diminished amylose content invariably corresponds to heightened starch transparency (Tester & Karkalas, 2001).

With accelerating social development and life expectancy, the demand for frozen foods has witnessed a discernible upsurge. Investigating the freeze–thaw stability of starch will help to promote the optimization of industrial production conditions for related products. The freeze–thaw precipitation rate of starch was significantly increased by nitrogen application, emblematic of a concomitant diminution in freeze–thaw stability, as depicted in Figure 3.

Intriguingly, the water precipitation rate of nSM surpassed that of wSM. Corroborating extant research, a negative correlation between amylose content and starch freeze–thaw stability has been underscored. Notably, cereals such as waxy foxtail millet and sorghum, characterized by amylose content, exhibit superior freeze–thaw stability (Arunyanart & Charoenrein, 2008), with findings congruent with the outcomes of the present inquiry.

#### 3.4.3 Water solubility and swelling power

**FIGURE 4:**
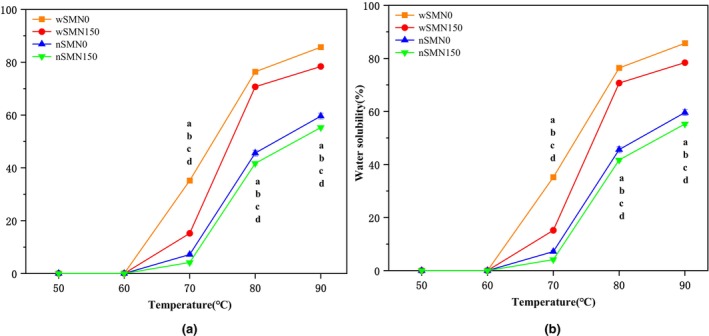
Water solubility (a) and swelling power (b) of wSM and nSM under nitrogen treatment at 50, 60, 70, 80, and 90°C. Different lowercase letters denote statistical differences between treatments at the 0.05 level in a column for (top to bottom) wSMN0, wSMN150, nSMN0, and nSMN150.

Solubility and swelling represent pivotal measures for discerning the extent of the interplay between amorphous and crystalline regions of starch chains and are important indicators of starch hydration capacity. As shown in Figure 4, the solubility and swelling degree increased significantly with the increase in temperature, and reached the maximum at 90°C. Solubility and swelling were significantly reduced after nitrogen application, and the peak values in two varieties were consistently observed under the control group (N0). The solubility and swelling of starch are influenced by multifarious factors, with amylose and amylopectin being the most prominent (Huang et al., 2015). Amylose has an inhibitory effect on the dissolution and swelling of starch granules. Nitrogen fertilization will also inhibit the swelling of starch and afford structural fortification to the starch granules (Syahariza et al., 2010). Our research showed that under nitrogen fertilizer application, the content of amylose in proso millet increased, while the solubility and swelling ability decreased. This mirrors the findings by Zhu et al. (2017), who reported an augmentation in the water solubility and swelling potency of rice starch in response to heightened nitrogen levels. Similarly, Li et al. (2013) documented an improvement in wheat starch's swelling capability after nitrogen application. On the contrary, our study emphasizes the significant reduction in water solubility and swelling potency of proso millet starch consequent to nitrogen treatment. These divergent outcomes may be ascribed to disparities in crop genetics and nitrogen dosages. The solubility and swelling of wSM starch are significantly higher than nSM, which may be attributed to the size and morphology of starch grains, molecular weight, straight/branched chain ratio, branching degree, molecular structure of branched starch, and other components like protein and lipid (Uarrota et al., 2013).

### 3.5 Correlation analysis

**FIGURE 5:**
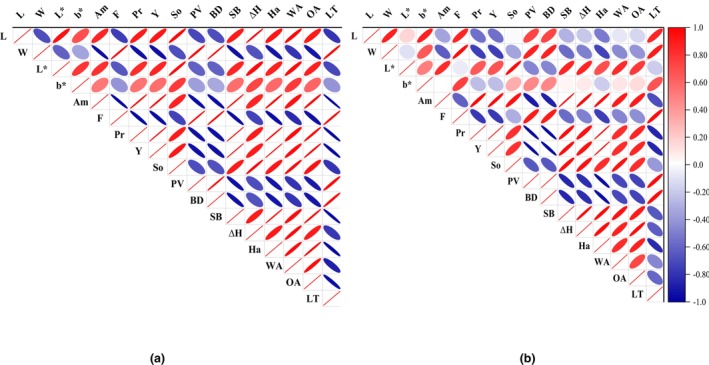
Correlation analysis of cooking quality and starch properties of wSM (a) and nSM (b) under nitrogen treatment. ∆H Enthalpy value; Am, Amylose content; BD, Breakdown value; F, Crude fat content; Ha, Hardness; L, Length; LT, Light transmittance; OA, Absorption capacities of oil; Pr, Protein content; PV, Peak viscosity; SB, Setback value; So, Soluble solid; W, Width; WA, Absorption capacities of water; Y, Yield.

The correlations among appearance quality, nutritional quality, cooking quality, and starch properties of proso millet between the control group (N0) and nitrogen treatment (N150) were analyzed by Pearson correlation analysis (Figure 5). Among these different indicators, positive and negative associations can be distinguished. Specifically, the amylose content of proso millet showed a significant positive correlation with the SB, ∆H, water, and oil absorption capacity, but it bore a pronounced antagonistic link with the PV and BD. Meanwhile, the protein content was positively correlated with the SB, ∆H, water, and oil absorption capacity. There were some differences in correlation coefficients between waxy and non‐waxy proso millet, which may be related to their genetic background differences.

## 4 CONCLUSION

Nitrogen application significantly affected the cooking quality and starch properties of proso millet. In comparison to the control group (N0), the protein content, amylose composition, and yield of proso millet increased significantly, which made proso millet easier to boil into porridge. The FV, SB, To, Tp, and ∆H of proso millet powder increased significantly, while the PV, BV, and BD decreased. The adhesiveness of proso millet rice decreased, and the texture was harder. Accompanying this, the water absorption and oil absorption of proso millet starch increased, while solubility and swelling power decreased. There were significant correlations among the appearance quality, nutritional quality, cooking quality, and starch properties of proso millet. In general, these results provide a new basis for the development of multi‐food proso millet and lay a foundation for the proliferation of high‐quality proso millet cultivation practices.

## AUTHOR CONTRIBUTIONS

**Hongyu Liu:** Conceptualization (equal); data curation (supporting); formal analysis (lead); software (lead); validation (equal); visualization (lead); writing – original draft (lead); writing – review and editing (lead). **Beibei Liu:** Data curation (equal); formal analysis (equal); investigation (supporting); methodology (equal). **Haolu Zhou:** Investigation (equal); methodology (equal). **Yinghui Huang:** Investigation (equal); methodology (equal). **Xiaoli Gao:** Conceptualization (equal); funding acquisition (equal); resources (equal); supervision (equal); writing – review and editing (equal).

## FUNDING INFORMATION

This research was funded by Green Technology Integration and Demonstration of High Yield and Benefit of Main Minor Grain Crops of Shaanxi Province, grant number “2023‐ZDLNY‐06”.

## CONFLICT OF INTEREST STATEMENT

The authors declare no conflict of interest.

## Data Availability

The data that support the findings of this study are available on request from the corresponding author. The data are not publicly available due to privacy or ethical restrictions.
